# Hazard Perception, Presence, and Simulation Sickness—A Comparison of Desktop and Head-Mounted Display for Driving Simulation

**DOI:** 10.3389/fpsyg.2021.647723

**Published:** 2021-04-22

**Authors:** Sarah Malone, Roland Brünken

**Affiliations:** Department of Education, Saarland University, Saarbrücken, Germany

**Keywords:** virtual reality, head-mounted display, driving experience, presence, simulation sickness, hazard perception assessment

## Abstract

Driving simulators are becoming increasingly common in driver training and assessment. Since virtual reality is generally regarded as an appropriate environment for measuring risk behavior, simulators are also used to assess hazard perception, which is considered to be one of the most important skills for safe driving. Simulators, which offer challenges that are indeed comparable to driving in real traffic, but at a very low risk of physical injury, have the potential to complement theoretical and practical driver trainings and tests. Although configurations and fidelity differ considerably between driving simulators, studies comparing the impact of their distinct features on driving performance and test validity remain rare. In this context, prior research demonstrated that a wider field of view (three monitors compared to a single monitor) led to earlier speed adjustments in response to potential hazards**—**especially for experienced drivers. The wider field of view was assumed to cause the drivers to be more present in the virtual world, which in turn provoked more natural scanning of the road and therefore, earlier hazard detection in experienced drivers. Research on spatial presence in other contexts support this assumption. The present experiment investigated whether this effect could be enhanced by an even more immersive presentation technique for driving simulation: a head-mounted display (HMD). Moreover, we studied the interplay between display mode, sense of presence and simulation sickness. Eighty experienced and less experienced drivers completed six simulation-based hazard perception scenarios, which were displayed either *via* a triple-monitor set-up or an HMD. Results indicate that the experienced drivers showed very similar driving and risk behavior as the inexperienced drivers in both experimental conditions. However, there were significant differences between the two display conditions. The use of an HMD resulted in a clearer and more abrupt speed reduction, more virtual presence, and a higher degree of simulation sickness. However, the interrelation between these three variables could not be conclusively clarified in the present study and thus represents a research aim that could be addressed in future studies.

## Introduction

Technology-based simulations are well-liked tools for entertainment, learning, and research in cases in which performing the corresponding experiment or training in real-life would be too expensive, too dangerous, or even impossible. Simulations often comprise virtual reality (VR) features, such as stereoscopic view provided by head mounted displays (HMDs), and in- and output devices that allow for a natural interaction with the simulation (e.g., force feedback steering wheels and seats in driving simulation). In recent years, VR simulations have become affordable even for private use and therefore, enjoy great popularity (e.g., Stanney et al., 2020). However, the expected advantages of VR-based simulations are not restricted to increasing joy or motivation; VR applications are particularly interesting for assessment purposes. de-Juan-Ripoll et al. (2018) argued that VR is particularly useful in the assessment of risk behavior due to its immersive characteristics and its ability to collect real-world behavioral metrics. As a result, VR assessment is exceptionally ecologically valid. Notably, the more authentic a VR experience, the more valid the inferences about real-life behavior that can be drawn from the behavior in the simulation.

Driving is a domain in which the assessment of risk behavior is particularly important. Therefore, simulators are considered useful to investigate driving-related high-level tasks that would pose a high risk of collision to the driver if encountered in a real environment. Consequently, driving simulators are suitable for hazard perception research and assessment, as they focus on driving behavior in potentially dangerous situations in traffic. The term “hazard perception” refers to a driver’s sensibility to recognize potential risks on the road ahead.

There are multiple methodologies to measure drivers’ hazard perception (Moran et al., 2019). In conventional hazard perception tests, participants watch several videotaped or computer-animated driving scenarios and are instructed to press a response button or touch the screen anytime they see an upcoming hazard. Most often, response times are recorded as indicators of hazard perception performance. Performance in hazard perception tasks has been found to be related to driving experience (e.g., Quimby and Watts, 1981; Borowsky et al., 2009, 2010; Smith et al., 2009; Scialfa et al., 2011, 2012; Malone and Brünken, 2016) and past and future traffic crash occurrence (e.g., Pelz and Krupat, 1974; Quimby and Watts, 1981; Quimby et al., 1986; Hull and Christie, 1993; McKenna and Horswill, 1999; Darby et al., 2009; Horswill et al., 2015). Taken together, research has confirmed, that hazard perception tests allow for a valid assessment of a skill that is crucial for safe driving.

Although hazard perception skills can be measured very well by this rather simple assessment technique, clearly, more is required to handle an actual road hazard than simply pushing a button in time. Grayson et al. (2003) noted four interacting components that comprise a driving-related risk behavior model: hazard detection, threat appraisal, action selection, and implementation. One drawback of the conventional video-based hazard perception tests is that they are restricted to measuring performance at the detection and appraisal levels, with both components even combined into a single response time for each hazard (Huestegge et al., 2010; Malone and Brünken, 2014). In contrast, driving simulation allows the assessment of the actions the driver chooses to avoid or deal with a dangerous situation (action selection), as well as whether they are able to perform these actions safely (implementation). Depending on the nature of a traffic situation, different behavioral responses aiming at collision avoidance may be a manifestation of hazard perception. Many situations require early speed reduction, merging into a suitable gap or, in some cases, accelerating to leave a hot spot as quickly as possible. However, ultimately, all actions that serve to avoid collisions could be considered expressions of hazard perception. It has been demonstrated that these actions adapt and become more fine-tuned as a result of perceptual-motor development and experience (for gap acceptance see, for example, Plumert et al., 2011).

The use of driving simulators for hazard perception research has been discussed from time to time, revealing specific benefits and disadvantages of this approach (e.g., Chan et al., 2010; Underwood et al., 2011). Among the advantages of simulators for hazard perception measurement is that they provide highly standardized tasks that can be repeated as many times as required. In contrast to filming real traffic scenarios, creating simulated scenarios is independent of existing structural conditions, current traffic, and weather. Moreover, in contrast to video-based approaches, driving simulations allow the investigation of driving as the complex task that it is, since simulations are assumed put more realistic demands on the participants. As a result, driving behavior metrics collected in a simulator might serve as good predictors for driving in real traffic. For research, they can serve as practicable supplements to the observation of drivers’ real on-road behavior, as observed on test routes or by evaluating daily driving in naturalistic driving studies (e.g., Klauer et al., 2006; Barnard et al., 2016).

Due to the evident ecological validity of simulations and their obvious benefits, the number of hazard perception studies featuring a driving simulator is growing (Crundall et al., 2010, 2012; Shahar et al., 2010b; Underwood et al., 2011; Borowsky et al., 2016; Tagliabue et al., 2017, 2019). However, some authors doubt that results from driving simulators can be generalized to the real world. Evans (2004) pointed out that unless simulators can measure how well a person is theoretically able to perform various driving tasks, simulator behavior does not necessarily predict what they will choose to do while actually driving on the road. However, for visual search behavior, it could be shown that the results of simulator studies match those of on-road studies. One explanation for why the hazard perception of inexperienced drivers is worse than that of experienced drivers is deficient visual search behavior in inexperienced drivers (Mourant and Rockwell, 1972). Simulator studies have replicated these findings from on-road studies, showing that inexperienced drivers failed to adapt their visual scanning behavior to different driving conditions (Crundall and Underwood, 1998), failed to monitor areas where a hazard was not yet present but was likely to emerge (Pradhan et al., 2005), and were more susceptible to distractions (Chan et al., 2010). In their review, Underwood et al. (2011) recapitulated empirical evidence for the comparability of hazard perception measurement in driving simulators, in conventional hazard perception tests, and on the road. They revealed similar experience-related search patterns for the three assessment techniques under investigation. Specifically, hazard perception, risk anticipation, and attention maintenance tasks in simulators seem to pose comparable demands on the driver’s visual scanning as real road hazards and video-based tasks do. Underwood et al. (2011) ultimately recommended simulators for hazard perception assessment, as they allow standardized and authentic tasks to be set in an ethically responsible manner.

However, the authors did not amplify one important fact about driving simulation: simulators differ in fidelity (the degree to which the simulation matches the conditions of the actual task) (e.g., Alessi, 1988). Display resolution, sounds, features of the hardware devices used, and interactions with the simulation, for example, can be more or less true to life. However, high-fidelity does not always have a fostering effect. Alessi (1988), for example, pointed out that higher fidelity also involves higher complexity, leading to higher workload, which can hamper learning and performance, especially for inexperienced trainees. Although simulation fidelity is an important issue in simulator development, empirical comparisons of driving simulator configurations are rare. Rosey and Auberlet (2014) investigated the impact of the kind of simulator [desktop (lower fidelity) vs. fully instrumented vehicle cabin (higher fidelity)] on driving behavior parameters using two identical driving situations (rural intersections). They continuously recorded the lateral car position, speed, and gas pedal use and compared variability measures of all three parameters. The average speed was higher in the desktop condition, whereas no main effect of configuration was found for lateral position and gas pedal use. The variability in lateral position was higher for the desktop simulator. The authors’ interpretation of the results was that in the full cab, participants paid more attention and adapted their behavior more to the situations. They cited greater immersion in the full cab as a reason for the more attentive behavior. Rosey and Auberlet (2014) compared two simulators that differed substantially in their degree of fidelity. In contrast, Shahar et al. (2010a) and Alberti et al. (2014) decided to vary only one feature of the visual display. In 2010 the former research group investigated the effect of an increased field of view on hazard perception performance in a conventional video-based hazard perception test by comparing a narrow-view (one screen) to a wide-view (three screens) condition. Two conflicting hypotheses were set. First, a wider field of view provides more information and therefore increases mental load, which results in lower performance in the hazard perception test. Second, if a wider view results in more short fixations, but with each long enough for complete processing, the wide-view condition will result in higher hazard perception performance. The main result of the study was that participants benefited from the wider view, which manifested in shorter average response times in the wide-view condition even if the side screens did not contain any hazard-relevant information. According to the authors, this result particularly supports the hypothesis emphasizing the more realistic experience as a reason for better performance. Later, Alberti et al. (2014) conducted a study to replicate the previous findings on the effects of a wider view in a simulation-based hazard perception test. By widening the provided field of view to a width corresponding to a natural view, the authors increased simulator fidelity. In addition to the width of field of view, they included driving experience as a second factor by comparing novice and experienced drivers. It was expected that the experienced drivers in particular would display safer driving behavior in the wide-view condition. The results indicated that fewer accidents occurred in the wide-view condition. Furthermore, experienced drivers drove more slowly in the wide-view condition than in the narrow-view condition, whereas no such difference existed for the novices. In the wide-view condition, the experienced drivers also slowed down earlier than the novices when approaching a potential hazard. Thus, the wide-view condition led to safer driving overall, but the experienced drivers made even more use of the wide-view condition than the novice drivers. As Underwood et al. (2011) stated that the validity of driving simulators can be evaluated by comparing the performance of drivers with different driving experience, one can conclude that a wide field of view preferentially supported experienced drivers and therefore, enhanced test validity.

Shahar et al. (2010a) and Alberti et al. (2014) explained their results by suggesting that a wider view provides more immersion (see also Allen et al., 2005), which results in a more realistic scanning of the environment and therefore better performance. As the inexperienced drivers’ scanning behavior on the real road was expected to be less developed than that of experienced drivers, the latter derived even more benefit from a wider view.

Although this explanation carries conviction, the presumed relations between width of field of view, immersion, visual search behavior, and performance were not investigated in the studies discussed. However, the response similarity approach delivers support for this explanation. IJsselsteijn (2004) stated that “it is reasonable to expect that as the fidelity of the displayed environment increases, responses to that environment will be increasingly similar to responses we exhibit to the same objects, agents or events in real environments” (p. 202). If the vividness of a simulation (graphical display, sounds, etc.) and the possibilities to interact with the mediated environment in a natural way are well marked, users might become deeply immersed into the displayed events. Therefore, technologies and techniques, such as VR simulations, that are designed to evoke and train real-world skills, often aim to be immersive (Wirth et al., 2007). Immersive technology is understood as a tool that dissolves the boundaries between the real and the virtually simulated world (Lee et al., 2013).

Thus, the level of immersion in a virtual environment is theoretically closely related to the degree of simulator fidelity. However, the circularity of this statement is not to be denied, since fidelity and immersion are not clearly separable in terms of definition. A possible solution for this issue might be to examine the relation between objectively detectable attributes of a simulation environment and the subjective experience of it as reported by users (*experiential fidelity*; Stoffregen et al., 2003). These thoughts lead to the consideration of another concept, namely virtual presence, which implies that the user feels part of the virtual environment and even has the impression of being spatially present amidst the displayed scenery. Steuer (1992) mentioned that individuals who experience presence perceive the mediated environment as real as their actual physical environment. The conclusion that they will also behave as if they were in a natural environment is quite reasonable. Hence, virtual presence could be expected to act as a mediator between objective indicators of simulator fidelity and related aspects of user behavior.

Presence can be assessed by subjective measures, asking users to judge or consciously explain their sensations or by objective measures such as behavioral indicators observed during simulation. Retrospective presence questionnaires are the most common of the subjective measurement approaches (e.g., Witmer and Singer, 1998). Objective behavioral measures, on the other hand, are rarely used, but can be expected to be less biased because behavioral responses are less consciously controlled by the user; they are displayed automatically. Furthermore, they can deliver continuous measures and are considered unobtrusive, which means that neither performance nor immersion are hampered or interrupted by the measurement. Examples of objective behavioral indicators for the measurement of presence are facial expressions, eye movements, and postural responses (e.g., body or head sway; Freeman et al., 2000).

There is empirical evidence for the width of field of view being a strong contributing factor to the experience of presence in simulations. Snow and Williges (1998) compared three levels of field of view for five different subtasks, including distance estimation and visual search. The level of field of view was positively related to presence, which in this study was measured by a subjective rating. Furthermore, the depth of presence was significantly positively related to performance in all subtasks.

Regarding visual display configurations, additional factors were found to affect presence. In an experiment conducted by Freeman et al. (2000), participants watched a video sequence filmed from a rally car speeding on a curvy rally track. The authors found that the participants’ self-rated sense of presence and simulator sickness and their body sway (recorded by a magnetic position tracker) were higher with stereoscopic view than with monoscopic view. Thus, a three-dimensional impression seemed to foster feelings of presence and natural postural responses.

As breaks in presence can be caused by sensory information from the real world (Slater and Steed, 2000), technologies that seal users off from reality are expected to be particularly suitable to evoke presence. Hence, the use of an HMD instead of desktop screens has proven to be beneficial for the evocation of presence. Shu et al. (2019) investigated the impact of HMD vs. desktop simulations in the context of earth-quake education. Learners in the HMD condition reported a higher sense of spatial presence than participants in the desktop condition, as well as higher self-efficacy toward earthquake preparedness. Nichols et al. (2000) could show on an objective level that reflex responses to stimuli emerging in a simulation were greater with HMD than with desktop. Moreover, the participants reported a higher sense of presence.

When people observe or interact with (virtual) moving stimuli, there is a certain risk for them to experience visually induced motion sickness (depending on the context: simulation sickness, cybersickness, VR sickness; e.g., Chang et al., 2020). Therefore, in addition to the sensation of virtual presence, simulator sickness is an important factor in simulated driving studies, as it can limit the use of advanced immersive technologies in general, such as virtual and augmented reality (Stanney et al., 2020). Simulator sickness manifests itself, for example, as nausea, headaches, eye discomfort, and drowsiness, which can develop during simulation and, if severe, can lead to quitting prematurely (e. g., Kennedy et al., 1993; Lawson, 2014; Chang et al., 2020). According to research, different factors and their interplay are considered causative for a person to develop simulator sickness. These factors relate to content aspects of the simulation (for example, the tasks to be performed during simulation) and to human factors (Chang et al., 2020; Curry et al., 2020). The frequency and severity of simulator sickness may also be, related to the type and quality of the device used for simulation (Stanney et al., 2020). Accordingly, Nichols et al. (2000) demonstrated increased simulator sickness when using an HMD instead of a desktop monitor.

Simulator sickness also appears to be related to how individuals behave during the simulation: research indicates that there are associations between simulator sickness and participants’ body movements. Body adjustments have been shown to be precursors for later onset sickness during the use of VR technologies (Chang et al., 2017; Stoffregen et al., 2017).

A mixed pattern of results exists in the research on the relation of simulator sickness and presence. In their review, Weech et al. (2019) investigated the relationship between the perception of presence and simulation sickness in different contexts of VR application. In all of the studies included, both variables were measured using subjective ratings. The results were mixed. Most of the correlations found were negative. In the context of driving simulations, however, a lack of correlation up to clearly positive correlations between presence and simulation sickness were found.

## The Present Study

Overall, research has suggested that simulators are suitable for hazard perception measurement. It can be assumed that higher simulator fidelity will be achieved using more immersive technology. This creates a greater sense of presence and thus more natural behavior, which might increase the validity of the measurement. The present study aimed in principle to demonstrate this relationship. As it is known from previous research that experienced and inexperienced drivers differ in their ability to recognize developing hazards on the road and that experienced drivers benefit more from a wide naturalistic view during simulated driving, we investigated whether a triple-monitor display or an HMD—the more authentic experience—is better suited to differentiate between these two driver groups in simulated driving, and how the display mode in general influences how the participants experience the driving simulation.

The main aim of the current study was to build upon the approach of Shahar et al. (2010a) and Alberti et al. (2014), who were able to enhance performance in hazard perception tests by evoking natural scanning of the road. The latter was achieved by providing a wider view of the traffic scenery, which the authors assumed would be closer to reality and thus elicit more presence in the users than a narrow view. Even though this explanation is grounded in sound theoretic assumptions and empirical results from other domains, the relation between display mode and presence could not be proven, because presence was not measured in the studies cited. Thus, to investigate the assumed relations, the current study included presence measures.

Whereas the previous studies used a triple-monitor set-up to create the wide-view condition, in the present study, we intended to offer the participants an even more realistic impression of the simulated environment by using an HMD, the Oculus Rift Development Kit 2. This VR headset provides stereoscopic view, which has been shown to be a presence-evoking feature. Furthermore, and in contrast to most HMDs used in the past, its displays are large enough to cover the entire field of view. Neither the frame of the goggle nor the real environment is visible when wearing the device, which further supports feelings of presence. Moreover, when wearing the HMD, the view is not restricted to the road ahead (including rear and side mirrors); participants can explore their entire surroundings by turning and inclining their heads.

Based on the previous results on the relations between hazard perception, naturalistic view, presence, performance, and simulator sickness we generated five hypotheses:

Hypothesis 1: Drivers will reduce their velocity when approaching a potential hazard area.

Hypothesis 2: Experienced drivers will outperform inexperienced drivers in hazard perception: Approaching a potential hazard area, they will reduce their velocity earlier and to a greater extent and they will be involved in fewer crashes.

Hypothesis 3: The effects assumed in Hypothesis 2 will be more accentuated for participants wearing an HMD compared to those using a triple-monitor set-up for simulation.

Hypothesis 4: Participants wearing an HMD will experience more virtual presence than participants using a triple-monitor set-up for simulation.

Hypothesis 5: Participants wearing an HMD will experience higher levels of simulation sickness than participants using a triple-monitor set-up for simulation.

## Methods

### Participants

A total of 80 individuals participated in the experiment, of whom 42 completed the HMD condition. However, three of the latter had to be excluded from the analyses because they felt too nauseated to finish the experiment. Of the remaining participants, 55% were female and their mean age was 24.01 years (*SD* = 4.55). All participants had normal or corrected-to-normal (color) vision.

The handling of the simulation was close to reality. In order to ensure that the drivers could master it and drive according to the traffic rules, it was necessary that all participants held a driver’s license. HP research typically compares groups of drivers with different levels of driving experience. The systematic review by Moran et al. (2019) revealed that the categorization of inexperienced and experienced drivers is inconsistent in hazard perception research: depending on the study, drivers who had held a driver’s license for 0.1 to 4.5 years were categorized as novices and drivers with 2 to 29.9 years of driving experience were considered experienced drivers. In most cases, the groups also differed in their mean age. As driving experience was a factor in the current experiment, participants were recruited to form two expertise groups (inexperienced and experienced drivers). To decide upon cut-off values regarding driving experience, accident research was taken into account. It has been demonstrated that drivers have a very high accident risk immediately after obtaining a driver’s license, which is reduced to only 10% of the initial risk after about 2 years of regular driving (Hansjosten and Schade, 1997; Mayhew et al., 2003). Within these first 2 years, the initial accident risk is reduced approximately exponentially (Sagberg, 1998). Therefore, for the group of experienced drivers, we recruited individuals who held their driver’s license for at least 2 years. To ensure that the drivers had gained practical driving experience, an additional requirement was that they had already driven more than 7,000 km since licensing. The group of inexperienced drivers included those who had driven a maximum of 5,000 km and held their driver’s license for less than one and a half years and those who had had their driver license for more years, but were infrequent drivers (<5,000 km driving experience overall). Drivers who had stated during a preliminary telephone interview that their driving experience was intermediate were not invited to participate in the experiment.

Overall, 48 participants reported that they regularly engaged in video gaming for 1 to 66 h per week (70% for less than 10 h), and 11 played driving simulation games. Six of them used a racing wheel for gaming, while only two used an additional pedal set. Whereas some of the participants had already used the Oculus Rift or a comparable HMD (17%), none of them owned such a device or used it for gaming.

In order to assess the physical presence of the participants in the simulation, the participants were filmed. However, not everyone consented to the video recording. Thus, analyses that refer to the physical presence of the drivers (head sways) only include data from 52 participants.

### Apparatus

The experiment was conducted to compare two display modes for simulation-based hazard perception tasks: an HMD and a desktop set-up with three monitors. For the HMD condition, we used the Oculus Rift Development Kit 2 to display six driving scenarios. The display resolution was 960 × 1,080 pixels per eye. The Oculus Rift provides a 100° field of view (nominal). The triple-monitor set-up comprised three 22″ PC monitors, which were arranged in a half-circle on top of a table to provide a nearly 180° field of view to the participants. The overall screen resolution was 5,040 × 1,050 pixels.

**FIGURE 1:**
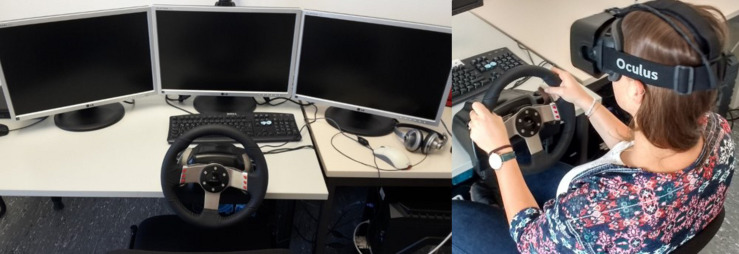
Simulator set-up in the triple-monitor condition and participant in the HMD condition.

Apart from the applied displays, both conditions used the same hardware devices, software, and instructions. Engine noise and navigation system-like audio instructions were provided *via* headphones. A Logitech G27 Racing Wheel device with a force feedback steering wheel and a pedal set with brake and gas pedal were used. Though the steering wheel and pedals were fixed, the chair was adjustable to the participants’ individual preferences. Automatic transmission was used. A high-performance desktop PC (Alternate BTO System) ran the simulation in both conditions (see Figure 1).

Throughout the entire experiment, the participants who had given consent were filmed from the front by a camcorder, which was placed on a tripod.

### Simulated Driving Scenarios

For both conditions, we used the same 3D database of the OpenDS driving simulator^1^. The participants drove six short simulated routes (each about 3 min, depending on the drivers’ average speed). The scenarios were derived from animations that were part of a hazard perception test that had already been pretested in preceding studies (Malone and Brünken, 2019). To assess hazard perception performance during simulated driving, one must interpret the behavior of drivers. To facilitate this, we selected for this study only scenarios in which an early adjustment of speed (by braking or releasing the accelerator), represents a particularly good indicator of hazard perception. In all scenarios, therefore, it was ensured that the driver approached the hazardous area from the front and that it was already possible to anticipate from a distance that a hazardous situation could develop. Accordingly, it could be counted as an expression of hazard perception if the driver reduced his speed by braking or releasing the accelerator.

Because Crundall et al. (2012) reported that experienced drivers outperformed less-experienced drivers to a greater extent if the hazards were latent or obscured but predictable, all scenarios contained hazards that could be anticipated from afar (at approximately 50 m) due to the presence of precursors. Moreover, the precursors appeared close to the center of the visual field (on the middle monitor in the triple-monitor set-up) to ensure that participants in both conditions had the same chance to spot the hazard clues regardless of their ability to see or scan the rest of the simulated environment. A short description of the contents of the scenarios and example images taken from the animations are shown in Table 1.

**TABLE 1:** Description of the six simulated driving scenarios.

No.	Scenario description	Required driver behavior	Image from animation
1	Driver approaches a stop area into which a train is entering. Driver ahead brakes because a person is approaching to cross the crosswalk further ahead to get the train.	Identify train stop as a critical area; see the approaching person, anticipate her intention toward crossing the road and reduce speed promptly.	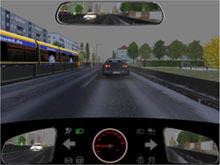
2	Drive on inner-city street, car ahead approaches slower female cyclist who is visible early from afar or later only through the other car’s windshield.	Driver recognizes early that there is a cyclist ahead and monitors her through the windshield of the car ahead, reduces speed and is ready to brake.	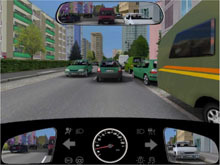
3	Drive on a country road, driver drives straight ahead a long distance with little traffic toward a curve that is difficult to overlook. In the middle of the bend, the driver encounters a very slow tractor on the same lane that was hardly recognizable before.	The driver recognizes the danger of a blind bend, reduces speed and is ready to brake.	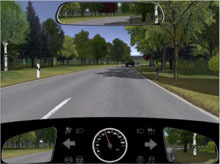
4	Driver follows two vehicles, while a car is approaching from behind. Latter starts to overtake all the three cars but has to stop the overtaking procedure because of oncoming traffic. Reeves directly in front of the test driver.	Driver monitors oncoming traffic and rear-view mirrors during driving and therefore anticipates the potential conflict, reduces speed and is ready to break and leave a gap for the overtaker to reeve in.	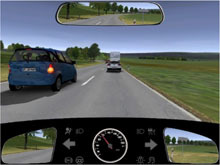
5	Driver approaches intersection area intending to turn right; in doing so, the driver must give priority to a male cyclist who is driving straight across the intersection to the right of the driver	The driver observes the cyclist from the start and does not forget him even when the cyclist is in the blind spot. Driver reduces speed early and slows down to let the cyclist pass.	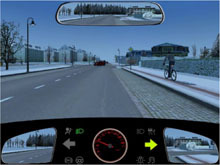
6	Drive in an outskirts residential area with vehicles parked alongside the right side. One of the parked vehicles flashes briefly and shears out onto the road in front of the driver	Driver recognizes the hazard of densely parked vehicles as soon as he approaches, slows down his speed, observes the parked cars and is ready to brake.	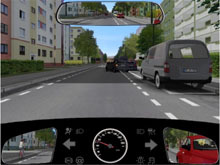

Using audio instructions over headphones, the participants were navigated through the scenarios to reach the potential hazard area.

The actions of other involved vehicles, pedestrians, or traffic lights were controlled using several invisible triggers. It was thereby ensured that the resulting scenarios were very similar for all participants and that they were all brought into a standardized potential hazard scenario at the end of each scenario, regardless of how fast they had driven approaching the hazard area (within certain limit, i.e., when the drivers stopped or drove very slowly after having passed the last trigger).

### Questionnaires

#### Demographics, Driving Habits, and Gaming

A computer-based questionnaire was administered to obtain demographic data (age, sex); information about driving experience (total kilometers driven, year of successful driving test, average weekly driving); and information about gaming behavior in general (average weekly hours), games involving driving simulations, and devices used for gaming.

#### Presence

The short versions of five subscales of a spatial presence questionnaire (MEC-SPQ; Vorderer et al., 2004) were used to assess the perceived presence of the participants. Each subscale was composed of four statements. A Likert scale from one (“I do not agree at all”) to five (“I fully agree”) was used to mark an answer. The subscale Attention Allocation measures to what extent the participants concentrate their attention on the virtual environment (example item: “I devoted my whole attention to the simulation”). The subscale Spatial Situation Model includes items referring to the perceived quality of the spatial situation model built by the participant during the simulation (example item: “I had a precise idea of the spatial surroundings presented in the simulation”). Self Location measures whether the participants had the impression of actually being in the virtual environment (example item: “I felt as though I was physically present in the environment of the presentation”). The subscale Higher Cognitive Involvement is composed of items regarding the intensity of participants’ thoughts about the virtual environment (example item: “The simulation activated my thinking”). The items of the subscale Suspension of Disbelief measure to what extent the participants minded errors and contradictions of the simulation (example item: “It was not important for me whether the simulation contained errors or contradictions”). The answers were recoded if necessary and summed across the subscales to generate a total presence score.

#### Simulator Sickness Questionnaire

The Simulator Sickness Questionnaire (SSQ; Kennedy et al., 1993) was administered to assess the severity of participitants’ health impairment after the simulation compared to their level of sickness before the simulation. The SSQ consists of a list of 16 symptoms (e.g., fatigue, eyestrain) that are associated with simulator sickness. Participants rated the severity of each symptom on a four-point scale (None—Slight—Moderate—High). The total score represents the overall sickness and is computed using a weighting procedure.

### Driving Behavior and Objective Presence Measures

#### Driving Behavior and Performance

To assess driving behavior in general and hazard perception skills in particular, two objective indicators were recorded. Collisions were counted throughout each scenario, while risk anticipation was inferred by speed reduction when approaching a potential hazard. Driving speed was logged continously throughout each scenario. On the one hand, we wanted to record when drivers began to reduce their speed, but on the other hand, the profile of speed reduction when approaching a potential hazard area provides information about how well one is able to adjust one’s speed to the situation. The latter also seems to be influenced by how the simulation appears visually (e.g., ground texture) and whether this supports fine-tuning of the deceleration (Fajen, 2005). To be able to track deceleration, we implemented the approach used by Crundall et al. (2012); see also Alberti et al. (2014): Speed was recorded at four waypoints, which were located at 10 m intervals starting 40 m in front of the position where the hazard cue would appear.

#### Head Sway

The videos of the participants doing the simulated hazard perception tasks were analyzed by a rater regarding medilateral sway of the upper body and head. Using a protractor with reference drawings of upper bodies that were inclined to the left or to the right, the rater counted all movements that could be classified as head sways and totaled them for each participant.

### Procedure

The study was made known to volunteers through posters at the university. In the previous contact by email or telephone, the future subjects were divided into experienced and inexperienced participants based on their driving experience. Persons who reported an intermediate amount of driving experience were not invited to participate. After this preliminary contact, the subjects were already divided between two experimental conditions (triple-monitor or HMD) to ensure that the two groups of expertise were equally distributed. This was done for each expertise group alternately in the order in which they responded to the invitation emails. This procedure corresponds to a balanced random assignment.

The participants were asked one at a time to the driving simulation laboratory. After a brief welcome, subjects were informed about the general objectives of the study. In addition, they received an information letter on possible symptoms of simulation sickness and on recommended behavior after the onset of such symptoms. The participants then signed a declaration of consent to participate in the study and to the planned processing of their data. The participants first completed a computer-based questionnaire on demographic data and driving experience. Then they filled out the SSQ for the first time as a baseline measurement. Afterward, they were familiarized with the driving simulation apparatus and the respective visual device was explained to them. In order to practice the handling of the simulation software and the steering hardware, a version of the lane change task was carried out (10 distances of 3,000 m each) using the respective OpenDS application. Subsequently, the test persons received an explanation of their task in the hazard perception scenario by the experimenter by means of instructional illustrations. As soon as the participants felt prepared, the camera was started, the headphones for navigation were put on, and the hazard perception simulation was launched. The six scenarios were presented one after the other in random order with a 30-s break in between. After the simulation, the participants were asked to fill in the SSQ again and the MEC-PQ for the first time on paper. Afterward, they were paid a reimbursement of 10 Euro and were dismissed.

### Design

The study followed a 2 × 2 × 4 mixed design with the two between subject factors *expertise* (experienced vs. inexperienced drivers) and *visual display* (triple-monitor set-up vs. HMD). The repeated measure was *proximity to the hazard area*; driving speed was measured at four way points in each scenario.

In addition to driving speed, further measures were included, although not repeatedly. The average number of collisions during a simulated scenario, the average difference of driving speed between the first and the last measurement points in the scenario, the difference of simulation sickness scores before and after the simulation, the subjective presence score, and the number of head sways performed during simulation were used as further dependent variables to answer the hypotheses of the current research.

## Results

### Validation of Expertise Groups

The participants were selected in advance to form two expertise groups. In order to check whether the split into experienced (*n* = 41) and inexperienced drivers (*n* = 36) had been successful and to what extent the two groups differed beyond their driving experience, several comparisons were made between the two groups. Using Welch’s *t*-tests, we confirmed that the groups of experienced and inexperienced drivers differed in the total number of kilometers driven since receiving a license, *t*(40.01) = 3.48, *p* = 0.001, *d*_*Cohen*_ = 0.74, and in average weekly kilometers, *t*(71.22) = 4.47, *p* < 0.001, *d*_*Cohen*_ = 0.99. Moreover, a *t*-test indicated that the experienced drivers were significantly older than the inexperienced drivers, *t*(72) = 2.75, *p* = 0.008, *d*_*Cohen*_ = 0.65 (three participants refused to provide their age). A chi-square test was used to compare gender and expertise. All expected cell frequencies were over 5. The results indicated that there was no significant difference between the two expertise groups in the proportion of men and women, χ^2^(1) = 0.97, *p* = 0.325.

We also examined whether the distribution of experienced and inexperienced drivers across the two visual display conditions was balanced with respect to these variables. The two groups did not differ in terms of kilometers driven since licensing, *t*(39.63) = 1.07, *p* < 0.290, in weekly kilometers, *t*(76) = 0.63, *p* < 0.532, in age, *t*(72) = 1.74, *p* = 0.086, or in gender distribution, χ^2^(1) = 0.19, *p* = 0.666. These analyses indicate that there were no significant *a priori* differences among conditions.

Descriptive demographic data and data regarding indicators of driving experience can be retrieved from Table 2, where they are divided according to the factors *expertise* and *display condition*.

**TABLE 2:** Sample description: number, means (standard deviations), and percentages for parameters of driving experience and demographic data, separate for display conditions and expertise groups.

	HMD		Triple monitor	
	Experienced	Inexperienced	Experienced	Inexperienced
N	23	17	19	19
Mean driving experience (km)	55695,65 (49133,80)	1484,71 (1391,84)	125444,44 (225232,74)	2,217,78 (1,885,14)
Mean weekly driving (km)	170,09 (130,02)	47,59 (57,29)	216,32 (191,172)	63,21 (134,76)
Mean age (in years)	24.61 (4.35)	21.24 (3.27)	26.53 (5.58)	23.79 (3.77)
Gender (percent m/f /d) ^a)^	48/52/0	35/65/0	53/47/0	42/58/0

*^*a)*^m, male; f, female; d, divers.*

### Risk Behavior

A mixed analysis of variance (ANOVA) with the repeated measures factor *proximity to the hazard area* and the two between-subjects factors *expertise* and *visual display* was conducted to analyze the effects of these factors on driving speed. The Greenhouse-Geisser adjustment was used to correct for violations of sphericity. The corrected values (*F*, degrees of freedom and *p*) are used whenever effects of the repeated measures factor are reported. Descriptive data can be found in Table 3.

**TABLE 3:** Descriptive data for driving speed at the four way points for the two display devices and driver groups.

Proximity	Visual display	Driving experience	*Mean*	*Standard deviation*	*n*
40 m	Triple monitor	Inexperienced	60.12	12.49	19
		Experienced	57.20	8.57	19
		Overall	58.66	10.67	38
	HMD	Inexperienced	61.57	7.51	17
		Experienced	63.65	5.63	22
		Overall	62.75	6.51	39
	Overall	Inexperienced	60.80	10.32	36
		Experienced	60.66	7.76	41
		Overall	60.73	8.99	77
30 m	Triple monitor	Inexperienced	56.78	11.10	19
		Experienced	54.47	8.34	19
		Overall	55.62	9.75	38
	HMD	Inexperienced	58.93	8.51	17
		Experienced	60.93	6.21	22
		Overall	60.06	7.27	39
	Overall	Inexperienced	57.79	9.88	36
		Experienced	57.93	7.88	41
		Overall	57.87	8.81	77
20 m	Triple monitor	Inexperienced	52.23	12.11	19
		Experienced	50.61	7.54	19
		Overall	51.42	9.98	38
	HMD	Inexperienced	53.84	10.11	17
		Experienced	56.40	6.76	22
		Overall	55.29	8.36	39
	overall	Inexperienced	52.99	11.08	36
		Experienced	53.72	7.62	41
		Overall	53.38	9.34	77
10 m	Triple monitor	Inexperienced	49.49	12.05	19
		Experienced	47.65	6.70	19
		Overall	48.57	9.66	38
	HMD	Inexperienced	49.76	10.17	17
		Experienced	50.03	7.41	22
		Overall	49.91	8.60	39
	Overall	Inexperienced	49.62	11.04	36
		Experienced	48.93	7.11	41
		Overall	49.25	9.10	77

The mixed ANOVA revealed a large main effect of *proximity to the hazard area*, *F*(1.85, 134.80) = 199.98, *p* < 0.001, partial η^2^ = 0.73. In accordance with Hypothesis 1, the data indicate that participants reduced their driving speed when approaching the hazard areas. The ANOVA did not reveal a significant interaction effect of the factors *proximity to the hazard area* and *expertise*, *F*(1.85, 134.80) < 1. Therefore, the data could not confirm Hypothesis 2, which stated that experienced drivers would slow down earlier than inexperienced drivers before a potential hazard situation. Moreover, the triple interaction effect (*proximity to hazard area* × *expertise* × *visual display*) was not significant, *F*(1.85, 134.80) < 1. This result contradicts Hypothesis 3, in which it was assumed that the effect of *expertise* would be particularly evident when approaching a hazard if an HMD was worn during the driving simulation. There was not a significant main effect for *expertise*, *F*(1, 73) < 1, or for *visual display*, *F*(1, 73) = 2.86, *p* = 0.095, nor was there a significant interaction effect of these two factors, *F*(1, 73) < 1. It appears that neither of the two factors had a significant influence on the average driving speed.

**FIGURE 2:**
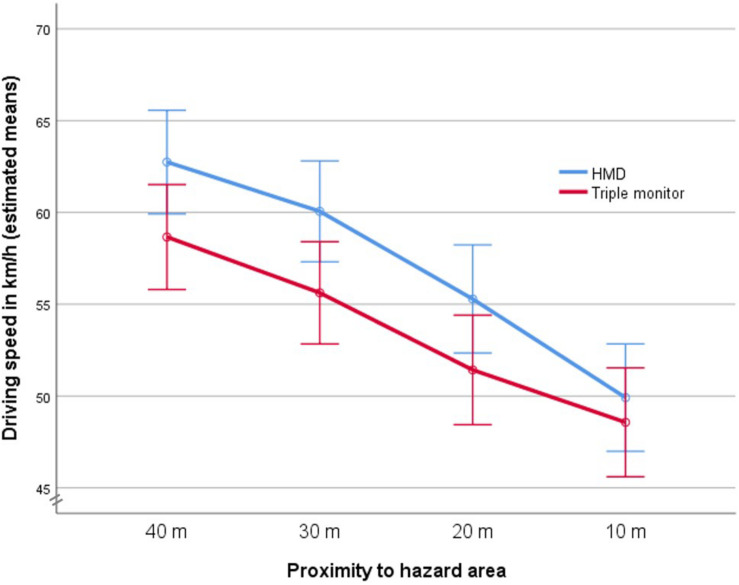
Approaching the hazard area in the two visual display conditions (error bars: +/− 2SE).

However, the mixed ANOVA revealed a significant interaction effect of the factors *proximity to the hazard area* and *visual display*, *F*(1,85, 134,80) = 3.64; *p* = 0.032. The descriptive data indicate a hybrid interaction: While participants in both display conditions showed a significant reduction in speed when approaching the hazard area, the drivers in the HMD condition slowed down more than the participants in the triple-monitor condition (see Figure 2).

To further examine the participants’ risk behavior, the dependent variables mean speed reduction and mean number of collisions during a scenario were included in further analyses (descriptive data can be retrieved from Table 4).

**TABLE 4:** Descriptive data for the reduction of driving speed and the number of collisions for the two display devices and driver groups.

Measurements	Visual display	Driving experience	*Mean*	*Standard deviation*	*n*
Speed reduction	Triple monitor	Inexperienced	10.27	7.41	19
		Experienced	12.03	7.47	19
		Overall	11.15	7.39	38
	HMD	Inexperienced	14.43	7.66	17
		Experienced	18.10	7.96	22
		Overall	16.50	7.94	39
Number of collisions	Triple monitor	Inexperienced	0.68	0.95	19
		Experienced	0.21	0.42	19
		Overall	0.45	0.76	38
	HMD	Inexperienced	0.65	1.06	17
		Experienced	0.96	0.98	23
		Overall	0.83	1.01	40

An ANOVA was performed to analyze the effects of the factors *expertise* and *visual display* on the dependent variable mean speed reduction from waypoint 1 to waypoint 4. The results of the ANOVA revealed a large main effect of the factor *visual display F*(1, 73) = 8.57, *p* = 0.005, partial η^2^ = 0.105, that confirms the results of the mixed ANOVA: In the HMD condition, a stronger speed reduction took place in view of an approaching danger. No main effect of the factor *expertise* was found, *F*(1, 73) = 2.41, *p* = 0.125, nor was a significant interaction between *visual display* and *expertise*, *F*(1, 73) < 1. A second ANOVA examined the effects of the factors *expertise* and *visual display* on the mean number of collisions during a simulated driving scenario. Neither a main effect of *visual display*, *F*(1, 73) = 3.08, *p* = 0.083, nor a main effect of *expertise*, *F*(1, 73) < 1, nor a significant interaction, *F*(1, 73) = 3.76, *p* = 0.056, could be found. The descriptive data show that the number of collisions was very low in all conditions.

### Virtual Presence

In a next step, we analyzed whether the type of visual display affected participants’ virtual presence. Since the objective and subjective measures and the associated measurement errors cannot be assumed to be uncorrelated, two unpaired *t*-tests were computed. Applying Bonferroni correction, the global alpha value was lowered to 0.025 in order to account for multiple comparisons. Only 52 participants agreed to be videotaped during simulation (*n* = 27 in the HMD condition). Therefore, the following results only apply to a portion of the total sample. The two visual display conditions differed significantly in the total number of head sways during the simulation, *t*(50) = 2.84, *p* = 0.007, *d*_*Cohen*_ = 0.80. On average, drivers wearing the HMD exhibited more head sways (*M* = 6.11, *SD* = 2.86) than those in the triple-monitor condition (*M* = 3.92, *SD* = 2.69). As head sways are considered indicators of virtual presence, this result supports Hypothesis 4. However, the difference between the HMD condition and the triple-monitor condition was not observed for the subjective presence measure, *t*(76) = 1.02, *p* = 0.313, as scores for the MEC-SPQ (Vorderer et al., 2004) were comparable in the HMD (*M* = 72.05, *SD* = 11.09) and the triple-monitor conditions (*M* = 74.58, *SD* = 10.87).

The objective and the subjective virtual presence measures were not significantly correlated (*r*_*Pearson*_ = −07; *p* = 0.622). Moreover, the number of head sways did not correlate significantly with the two variables of driving behavior, speed reduction (*r*_*Pearson*_ = 0.17; *p* = 0.242), and number of collisions (*r*_*Pearson*_ = 0.11; *p* = 0.445). The same applies to the subjective presence rating, which did also not correlate to the parameters of driving behavior measured (speed reduction *r*_*Pearson*_ = −0.03; *p* = 0.770; number of collisions *r*_*Pearson*_ = −0.02; *p* = 0.891).

### Simulation Sickness

Further analyses were performed to investigate whether the two visual display conditions triggered different levels of discomfort indicating simulation sickness (SSQ score). An unpaired Welch’s *t*-test supported Hypothesis 5, as the conditions indeed evoked different levels of simulation sickness during simulation, *t*(64.24) = 3.52, *p* = 0.001, *d*_*Cohen*_ = 0.80. The descriptive data indicate that an increase in the SSQ score occurred only in the HMD condition (*M* = 61.95, *SD* = 104.56), whereas the participants in the triple-monitor condition reported no increase during the simulation (*M* = −6.35, *SD* = 62.46). However, the overall values on the SSQ were not very high, and only 7% of the participants in the HMD condition had to terminate the experiment early.

Furthermore, simulation sickness was not significantly correlated with the virtual presence indicator head sways (*r*_*Pearson*_ = −0.04; *p* = 0.802) but was positively correlated with the reported virtual presence in the MEC-SPQ (*r*_*Pearson*_ = 0.24; *p* = 0.038). In addition, simulation sickness was positively correlated with speed reduction (*r*_*Pearson*_ = 0.27; *p* = 0.018), but not significantly correlated with the number of collisions (*r*_*Pearson*_ = −0.10; *p* = 0.366).

## Discussion

The present study was conducted based on the assumption that immersive VR simulations trigger naturalistic risk behavior and are therefore suitable and ecologically valid assessment tools (de-Juan-Ripoll et al., 2018). This is particularly relevant for simulator-based hazard perception tests, as they are intended to measure an important skill for safe driving. We compared the driving behavior of experienced and inexperienced drivers, who each completed six simulated scenarios, each of which ended with a dangerous situation. Different display devices were used to present the simulation: half of the participants saw the traffic environment on triple-monitor set-up in a semicircle, whereas the others wore HMDs. Driving behavior and indicators of presence and simulator sickness were measured.

All driving scenarios were designed to end with a potentially hazardous situation that could be predicted due to precursors appearing approximately 50 m before the actual hazard area. Therefore, our first hypothesis was that participants would recognize these precursors and slow down as they approached the hazard. This hypothesis was confirmed by the data, which showed a clear gradual decrease of average speed for all six scenarios. This implies that, in general, the chosen scenarios and their implementation in the simulator were successful in that they included early detectable cues and therefore predictable hazards, which is the basis for good hazard perception tasks that discriminate between safe and unsafe drivers (Crundall et al., 2012).

The second hypothesis was concerned with the impact of the factor driving experience. Underwood et al. (2011) pointed out that the ability to differentiate among drivers with different levels of driving experience can serve as an indicator of simulator validity. Moreover, research in the domain of hazard perception has revealed that experienced drivers usually outperform novice drivers in hazard perception tasks, regardless of whether photos, videos, or driving simulations were used (e.g., Crundall et al., 2012; Malone and Brünken, 2016). The results of the present study did not confirm this hypothesis, as the experienced drivers did not reduce their velocity earlier than inexperienced drivers when approaching a hazard area, nor were they involved in fewer accidents during the simulation. These results demonstrate that either the chosen scenarios or the applied measures could not differentiate between the two driver groups. Therefore, these tasks cannot be assumed to be valid measurements of hazard perception. There are several reasons that may explain why the study could not replicate the results of previous research. First, the expert groups were not very different in terms of their driving experience. It is possible that many of the participants classified as inexperienced in the present study had already reached the stable safe driving level of experienced drivers. Furthermore, the scenarios were not intended for simulator-based hazard perception measurement but were derived from a video-based hazard perception test (Malone and Brünken, 2019). In the study by Malone and Brünken, the differences between experts and novices were revealed in the early detection of dangerous situations, which was indicated by a button press. It is possible that the experienced drivers in the current study discovered the potential hazards earlier than the inexperienced drivers, but that it was not necessary to take countermeasures at this point. By the time an actual speed adjustment was necessary, the inexperienced drivers may also have identified the hazard area. Future research could use eye-tracking to test the latter assumption.

The results of the study by Alberti et al. (2014) suggested that experienced drivers in particular benefit from a naturalistic field of view in hazard perception tasks. The authors found that experienced drivers outperformed novices more if the driving simulation was presented on three monitors instead of one monitor. The HMD used in the present study allows a surround view, which is even more similar to the visual field when driving on the road than a triple-monitor set-up. Therefore, as a third hypothesis, we expected the performance gap between experienced and less-experienced drivers to be greater in the HMD condition than in the triple-monitor condition. This assumption could not be confirmed in the present study, as the visual display did not affect the differences in driving behavior between experienced and inexperienced drivers in terms of how early or by how much the speed was reduced when approaching a potential threat or in terms of the number of accidents. This may be because the task was not sensitive enough to map the differences between the driver groups. However, it may also be due to the fact that the HMD on which the simulation was presented was not as realistic as expected. This assumption is partly supported by the results related to Hypothesis 4, discussed below. Another possible explanation is that the difference in the sensation of presence between the HMD condition and the triple-monitor condition was not as large as the difference between the one-monitor condition and the triple-monitor condition in the study by Alberti et al. (2014).

Based on results from previous studies (e.g., Shu et al., 2019), in Hypothesis 4, it was assumed that the participants would experience more virtual presence during the HMD simulation than during a simulation with the triple-monitor set-up. This hypothesis was confirmed for the objective presence measure (head sways), but not for the subjective presence measure. Moreover, these measures were found to be uncorrelated in the present sample, which indicates that subjective measures of presence are unlikely to be strongly related to postural adjustments. Moreover, the study revealed that the differences between the two visual displays were likely not particularly large in terms of the perception of presence. At the very least, they were not detectable by subjective measurement *via* the questionnaire used (Vorderer et al., 2004), as it was probably not sensitive enough to detect such small differences. The results may also have been due to the design of the study. The differences would likely have been more evident in the subjective measurement if the participants could have directly compared the two visual displays. The fact that the objective and the subjective presence indicators showed no relationship to one another is surprising, as it implies that these two metrics do not measure the same latent variable. This may be because the two differ not only in the fact that one is subjective and the other is objective-behavioral, but also in that one is a real-time measure while the other is a hindsight rating. Further research is needed to specify the relationship between objective and subjective measures in driving simulations. Furthermore, the results showed that both were uncorrelated with the measures of driving behavior. This argues against the assumption by Alberti et al. (2014) that increased presence mediates the influence of the visual display on driving behavior and further, our results challenge the widespread idea that presence has some important relation to performance. On the basis of the current study, however, one does not have to reject this assumption completely if one assumes that the difference in presence perception caused by the two visual displays was too small to have a significant influence on driving behavior.

Based on previous studies using driving simulators and different display modes, it was assumed that participants in the HMD condition would develop more simulation sickness than participants in the triple-monitor condition. This last hypothesis was confirmed in the present study. These results are therefore consistent with those of Freeman et al. (2000), who found that, compared to monoscopic view, stereoscopic view led to more simulation sickness and body sway. Simulation sickness and head sway were not correlated; however, there was a small positive correlation between simulation sickness and the self-reported presence score. This is consistent with the findings of the review by Weech et al. (2019), who found zero to medium-high positive correlations between simulations sickness and self-reported feelings of virtual presence in driving simulation tasks. However, it is likely that these higher levels of discomfort in the HMD condition compensated for any benefits of the more immersive technique and were responsible for the fact that the validity-enhancing effects of HMD were not evident in the present study.

The results of this study are mixed as far as the hypotheses are concerned. However, the study has produced additional results that were not anticipated. For example, regardless of their driving experience, the participants reduced their speed more drastically and abruptly in the HMD condition than in the triple-monitor condition when they were close to the hazard area. This could be an indication that they experienced the dangerous situations more vividly and therefore as more dangerous, which in turn indicates a higher sense of presence. Whether this assumption is true could be investigated in future studies by implementing the thinking aloud technique to have the participants report their intentions and feelings during simulated driving. According to Fajen (2005), however, one could also assume that the HMD was less suitable for visually supporting a smooth deceleration process. This assumption could be investigated, for example, by fine-tuned experimental studies in within-design.

Another open question is whether the behavior evoked by the HMD simulation or the triple-monitor condition is closer to real driving behavior when facing a hazard on the road. In-depth analyses of video data from naturalistic driving studies (e.g., Barnard et al., 2016) are required to identify similar scenarios and to qualitatively compare the behavior of drivers operating real vehicles with those in simulated scenarios.

A further unexpected finding was the relation between simulation sickness and driving behavior: simulator sickness was positively correlated with speed reduction. It is possible that there was a causal relationship: increasing discomfort could have caused participants to have difficulty with speed adjustment or even to intentionally slow down realizing that their driving skills were currently limited. However, this cannot be resolved by the present study and could be a fruitful field for further research.

The present study has several shortcomings that might limit the generalizability of its results. Two limitations are particularly striking. First, only a very small number of simulated traffic scenarios were used in this study. Normally, more than twenty traffic situations are used in studies with video-based hazard perception tests (e.g., Malone and Brünken, 2019). However, the development of standardized simulation-based hazard perception tasks is much more complex. Nonetheless, it can be expected that, especially in the study of simulation-based hazard behavior, it is important to present many scenarios, as standardization is only possible to a limited extent. The participants always encounter slightly different situations, as the simulation always has to adapt a little to the individual driving behavior. For future research in this area, a large number of scenarios should be developed and varied in a controlled manner in order to identify factors that constitute valid simulated hazard scenarios that can distinguish between safe and unsafe drivers. The second limitation is related to the length of the driving scenarios. In order to avoid high levels of simulator sickness, the driving scenarios used were relatively short. In each scenario, the participants only drove for a few hundred meters before the hazard area was reached. The next scenario followed after a short break. This procedure certainly helped to prevent strong discomfort and to keep the strain on the participants low, as the drop-out rate was relatively low in the current experiment. However, the short driving times and interruptions may also have made a sensation of presence less likely to develop. Further research should therefore include longer driving scenarios to ensure that the participants adapt to the virtual world.

## Conclusion

The present study has shown that the type of visual display used in driving simulation-based hazard perception tests influences driving behavior, virtual presence, and the development of simulation sickness. However, it has not yet been demonstrated how these factors actually interact and affect the validity of the assessment. Future studies should address this research gap and, in particular, explore how realistic driving behavior can be induced by driving simulation features.

## Data Availability Statement

The raw data supporting the conclusions of this article will be made available by the authors, without undue reservation.

## Ethics Statement

Ethical review and approval was not required for the study on human participants in accordance with the local legislation and institutional requirements. The patients/participants provided their written informed consent to participate in this study.

## Author Contributions

SM has developed the study design, conducted the research and the data analyses and has written the major part of the manuscript. RB has provided consulting support to SM during all phases of the research for this study and contributed several text parts to the introductory and discussion part of the manuscript. Both authors contributed to the article and approved the submitted version.

## Conflict of Interest

The authors declare that the research was conducted in the absence of any commercial or financial relationships that could be construed as a potential conflict of interest.

## Footnotes

1 https://opends.dfki.de/
